# Training Interventions Used in Postmenopausal Women to Improve Pelvic Floor Muscle Function Related to Urinary Continence—A Systematic Review

**DOI:** 10.3390/jcm14134800

**Published:** 2025-07-07

**Authors:** Magdalena Piernicka, Justyna Labun, Anna Szumilewicz

**Affiliations:** Faculty of Physical Culture, Gdansk University of Physical Education and Sport, 80-336 Gdansk, Poland; justyna.labun@awf.gda.pl (J.L.); anna.szumilewicz@awf.gda.pl (A.S.)

**Keywords:** Kegel muscles, postmenopausal women, pelvic floor muscle training, quality of life

## Abstract

**Background:** The aim of this review was to analyze training interventions used and their effectiveness in improving pelvic floor muscle function related to urinary continence in postmenopausal women. We then characterized the recommended pelvic floor muscle training programs used in experimental studies based on four training components: frequency, intensity, duration, and type of pelvic floor muscle exercise. **Methods**: For this purpose, we conducted a literature review of works published up until the end of 2024, available in the Web of Science, PubMed, MEDLINE, and SPORTDiscus with Full Text databases. We used the keywords “pelvic floor muscle”, “training”, and “postmenopausal women”. Initially, we identified 205 articles published between 1997 and 2024. Then, based on specific criteria, we qualified 15 for analysis. **Results**: Thirteen studies included only PFMT, while three of them combined PFMT with other physical activity. In two studies, training was conducted in the form of a virtual video game using a pressure platform. We have noted that researchers most often use a 1 h pad test, digital palpation, and surface electromyography to assess the function of pelvic floor muscles. In improving pelvic floor muscle function related to urinary incontinence, 14 out of the 15 analyzed studies showed improvement. In only eight of the fifteen articles, researchers characterized all components of the implemented PFMT that enable full replication of the training intervention. In four of the studies, only one of the required components, namely intensity, was missing. The recommended number of training sessions was 2 to 7 per week, on average 3 ± 2 (M ± SD). Training interventions lasted from 2 to 24 weeks, on average 10 ± 6 weeks. **Conclusions**: Regardless of the chosen form of training intervention, PFMT is an effective method in improving the function of pelvic floor muscles in postmenopausal women.

## 1. Introduction

Pelvic floor muscles have a functional impact on the quality of life of women of all ages. Their most important role is their supportive function, as they support the pelvic organs, including the bladder and uterus. The pelvic floor muscles maintain control over excretory function by regulating urination and defecation. For proper functioning of the excretory mechanism, it is important to be able to control both contraction and relaxation of the pelvic floor muscles [1,2]. Moreover, they are important in sexual function [3,4] and during the course of childbirth in women [5,6]. Pelvic floor muscle function disorders are associated with social isolation, low self-esteem, and a sense of discomfort, affecting deterioration of women’s quality of life [7].

Based on studies conducted in the USA, it was determined that stress urinary incontinence affects from 25 to 55% of women over 60 years of age, with the risk increasing with age [8]. Moreover, global epidemiological data indicate that UI affects approximately 25–45% of adult women. The frequency of occurrence resulting from changes observed in women after menopause makes this group particularly vulnerable to pelvic floor dysfunction, affecting approximately 50% [9]. Menopause is the period in a woman’s life when, for biological and genetic reasons, menstruation stops, and therefore reproductive function is lost [10,11]. These changes are a natural process of aging and manifest themselves most often in women aged 45 to 55. At this stage of life, numerous physiological changes occur, including a decrease in estrogen levels, leading to atrophic changes in the vaginal and periurethral tissues, and thus to muscle weakness. Hormonal changes and alterations in the urogenital system may lead to intensification of pelvic floor dysfunction [12]. Episodes of uncontrolled urine leakage most often occur as a result of increased intra-abdominal pressure during coughing, sneezing, jumping, lifting weights, laughing, or having sexual intercourse [1,13].

One of the most widely recognized non-pharmacological interventions in the prevention and treatment of pelvic floor muscle dysfunction is pelvic floor muscle training (PFMT). PFMT has its origins in the work of Arnold Kegel, who in 1948 introduced a series of exercises designed to strengthen the pelvic floor muscles and decrease urinary continence, especially in postpartum women. Initially, the exercises mainly consisted of consciously contracting and relaxing the pelvic floor muscles. Over the years, many researchers have worked on modifying the exercises based on the Kegel’s program, expanding the methods used along with the protocols in different groups of people and using additional tools [14,15]. Engaging in training that includes both strengthening and relaxation exercises can be a key factor in proper functioning of the pelvic floor muscles [1,2]. Moreover, depending on the intervention used, other therapies may also be included in conservative treatment, such as laser therapy and electrical stimulation [1,16], magnetic stimulation [17] or radiofrequency therapy [1]. To assess the function of the pelvic floor muscles, diagnostic tools and techniques are used to enable detailed assessment of their strength, tension, and coordination. The most frequently used methods include palpation, ultrasound, manometer, dynamometer, perineometer, and surface electromyography [18]. One of the methods used to increase the effectiveness of learning correct movement patterns during pelvic floor muscle exercises is the biofeedback method [19,20]. The use of biofeedback based on visualization of the neuromuscular activity of the pelvic floor muscles enables real-time monitoring of activities. In a 2021 literature review, Romeikienė and Bartkevičienė observed that greater training effectiveness was noted in groups in which the intervention included use of the biofeedback method [21]. The methods of PFMT used across studies show considerable variation, due to several factors: individualization of treatment according to patient needs and functional capacity, the absence of a universally accepted standardized protocol, differing therapeutic aims, and varying clinical settings. Moreover, technological advancements such as biofeedback, electromyographic stimulation, and virtual reality-based training have significantly influenced the delivery of PFMT interventions, enabling the development of new, innovative approaches. Therefore, the choice of PFMT methods in the reviewed studies is not casual, but rather grounded in current scientific evidence, clinical expertise, and the integration of modern therapeutic tools.

According to the American College of Sport Medicine guidelines for conducting various types of training, an important element of training effectiveness is the use of principles of describing the concept enabling the replication of the intervention. To implement the recommended program, one must have information about the basic training components in accordance with the FITT principle (frequency, intensity, time, and type of exercise). The frequency of training is determined in the number of training units per week. In the case of training the pelvic floor muscles, the intensity is usually determined in relation to the maximum contraction. Training time refers to a single training unit, expressed in minutes or number of repetitions/sets. Additionally, the total duration of the training program should be specified, specifying the number of training sessions or the number of weeks after which positive changes can be expected. The type of training should include a detailed description of the exercises, including the technique and position [22].

In this review study, we set two research aims. First, the focus was on analyzing training interventions used and their effectiveness to improve the function of the pelvic floor muscles related to urinary continence in postmenopausal women. Secondly, we wanted to characterize the recommended PFMT programs used in experimental studies based on four training components: frequency, intensity, time, and type of pelvic floor muscle exercises.

## 2. Materials and Methods

The review of the literature was carried out from September 2024 to December 2024 in the Web of Science, PubMed, MEDLINE, and SPORTDiscus with Full Text databases, using the keywords “pelvic floor muscle”, “training”, and “postmenopausal women”. We found 205 articles published in 1997–2024. The systematic review that we performed following the guidelines of the PRISMA (Preferred Reporting Items for Systematic reviews and Meta-Analysis) statement [23]; however, it was not registered in a review registry such as PROSPERO. We selected articles according to the following criteria for inclusion in the analysis: publication in English, conducting experimental research using pelvic floor muscle training in postmenopausal women with urinary incontinence, and characterizing at least one training component for the applied training intervention (frequency, intensity, volume and/or type of exercise). We adopted the following exclusion criteria for the study: publication in a different language, women in a different age group, women diagnosed with urinary tract diseases, patients after stroke, multiple sclerosis, and pelvic organ prolapse. We also excluded articles whose main purpose was to assess other aspects (e.g., assessment of sexual function, assessment of motivation, assessment of physical activity, etc.) and those that did not contain a description of the PFMT. We also excluded studies where the full text was unavailable and studies that were inconsistent with the research objectives or were not experimental studies. We qualified for analysis 15 articles that met the inclusion criteria for the analysis and did not meet the exclusion criteria. Duplicates were removed from the search results. Two of the authors screened the titles of studies using the inclusion/exclusion criteria. Studies with titles suggesting eligibility for inclusion had their abstracts reviewed. If the abstract indicated that the study might be eligible for inclusion, then the full text was retrieved for review. Full texts were retrieved and independently reviewed and analyzed by two investigators (MP and JL). In case of disagreement on whether a paper should be eligible for inclusion, a third investigator (AS) was available to make the final decision. No automation tools were used in this process.

To determine which studies were eligible for each synthesis, we created a table in Microsoft Excel summarizing the key characteristics of the included studies, such as the characteristics of the study group, the type of intervention used, the research tools used, and the effectiveness and characteristics of the training components in the intervention conducted. These characteristics were manually compared against the predefined criteria for each planned synthesis. Based on this comparison, studies were manually assigned to specific syntheses according to their relevance to and compatibility with the planned comparisons. No methods were used to explore potential causes of heterogeneity, as the number of included studies (n = 15) was insufficient to support reliable subgroup analyses or meta-regression.

**Figure 1:**
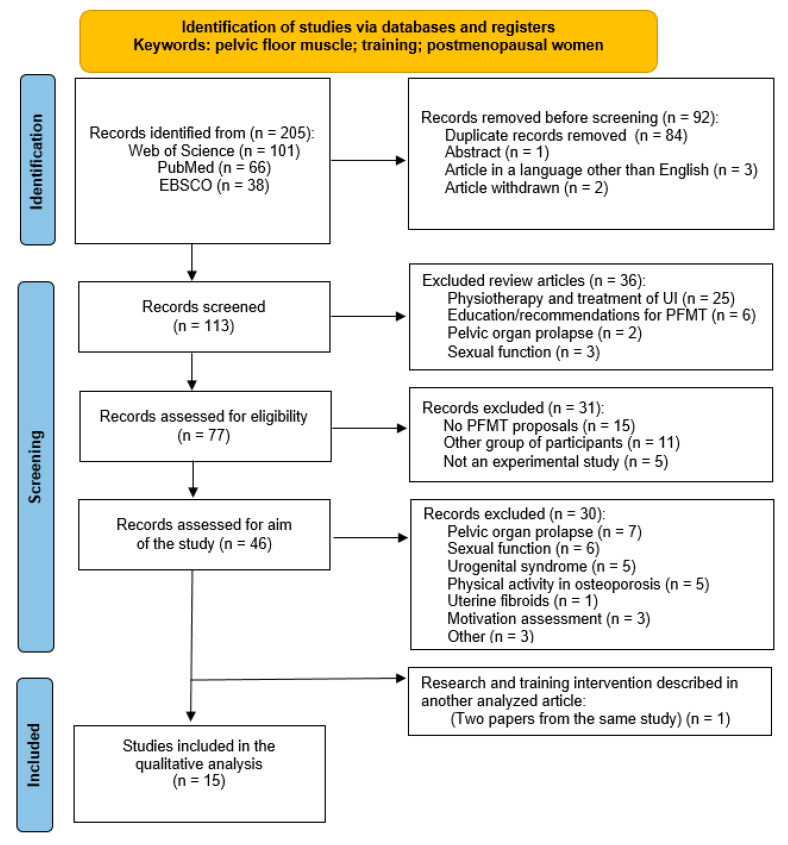
The process of qualifying research for analysis.

The detailed process of qualifying articles for analysis is presented in Figure 1.

This review included both randomized controlled trials (RCTs) and non-randomized controlled trial (NRCTs). Two independent reviewers assessed the risk of bias and methodological quality using the Cochrane Risk of Bias Tool for RCTs and the COSMIN checklist for NRCTs. For the included RCTs, the risk of bias was assessed using the Cochrane Risk of Bias 2.0 (RoB 2) tool, in accordance with the Cochrane Handbook for Systematic Reviews of Interventions [24]. The assessment covered five key domains: (1) bias from the randomization process, (2) deviations from intended interventions, (3) missing outcome data, (4) outcome measurement, and (5) selection of the reported result. Each domain was rated as “Low risk”, “Some concerns”, or “High risk”. To visualize the findings, the Robvis package in R was used to generate traffic light and summary plots, offering a clear overview of bias distribution across studies [25]. The COSMIN Risk of Bias checklist was used to assess methodological quality and potential bias in the included NRCTs, following the recommendations of Mokkink et al. (2020) [26]. This tool evaluates ten key domains related to patient-reported outcome measures (PROMs), such as content validity, structural validity, internal consistency, reliability, and responsiveness. Each domain is rated on a four-point scale: very good, adequate, doubtful, or inadequate. The overall risk of bias is determined using the “worst score counts” method, with non-applicable criteria excluded. Criteria marked as not applicable (NA) are excluded from the final judgment [26].

## 3. Results

A total of 895 postmenopausal women were examined in the 15 scientific articles qualified for analysis. We have presented a summary of analyzed articles in Table 1, including study groups, research tools, and achieved improvement results regarding pelvic floor muscle function (Table 1). All articles included in this systematic review are randomized controlled trials. In terms of the effectiveness of the training interventions applied, we observed statistically significant improvements in pelvic floor muscle function related to urinary incontinence in 93% of the analyzed studies [16,17,27,28,29,30,31,32,33,34,35,36,37]. In one study, no statistically significant changes were observed [38]. The EMG-based biofeedback method was used in three studies [16,31,34], whereas in two studies, biofeedback was used in the form of a virtual video game [33,39]. One study used sensory-motor biofeedback in the form of vaginal cones [27,28].

Only eight out of fifteen articles characterized all components of the implemented PFMT, which enables full replication of the research intervention [16,17,27,28,29,30,34,35,40]. Four studies provided all required information apart from specifying intensity [31,33,36,39]. In all studies in which intensity was determined (n = 8), maximum contraction of the pelvic floor muscles was recommended as a reference value. The frequency of training per week was provided in each analyzed study. The number of training sessions recommended from 2 to 7 per week, with on average 3 ± 2 (M ± SD). About half of the analyzed studies (53%) included information about undertaking a training intervention twice a week [17,27,28,29,31,33,34,39]. The duration of a single session was specified in eleven publications, specifying the number of repetitions and the number of exercise sets [16,17,27,28,29,30,31,33,34,35,36,40], while in three publications it was specified only in minutes [32,38,39]. The training interventions lasted from 2 to 24 weeks, on average 10 ± 6 weeks. Taking into account the training frequency and duration of the intervention, we noted that, in the analyzed studies, from 7 to 84 individual training sessions were conducted, on average 29 ± 26. Additionally, information on training progression was included in five studies [29,30,33,39,40]. In Table 2 we presented the components of the training programs used in the analyzed studies.

This study employed the RoB 2.0 tool to assess the risk of bias across the included randomized controlled trials (RCTs), with visualizations generated using the Robvis package. Among the included studies, one was classified as having a high overall risk of bias, while twelve were deemed to raise some concerns, and one study was assessed as low risk overall. Of the domains assessed, the highest number of studies with some concern was observed in the domain of bias due to the randomization process (n = 9), mainly due to insufficient information on the randomization procedures or allocation concealment. Similarly, seven studies raised concerns in the domains of bias in outcome measure (D4) and bias in selection of reported outcome (D5), concerns mainly related to lack of information on the blinding of outcome assessors and unclear reporting strategies.

**Figure 2:**
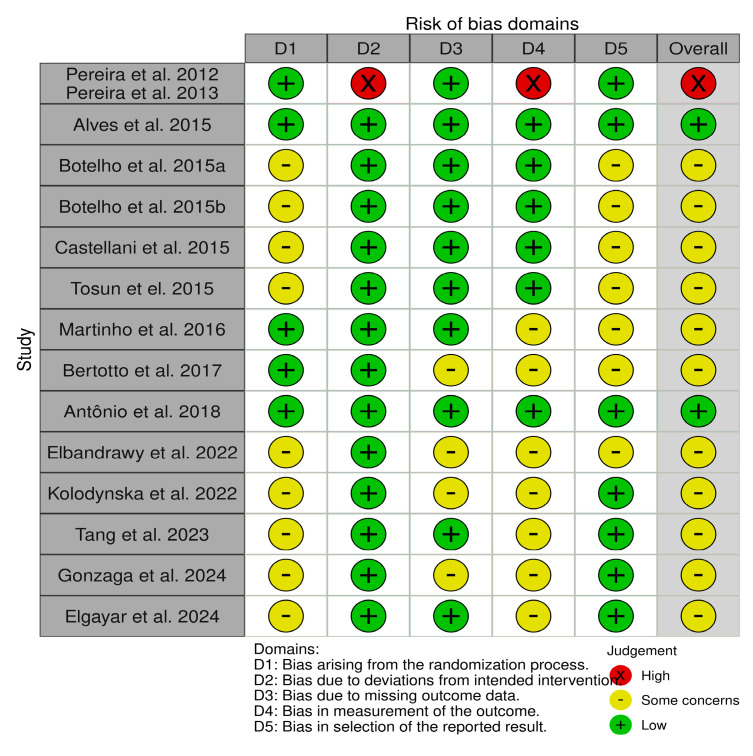
Risk of bias assessment of the included randomized controlled trials using the RoB 2.0 tool.

**Figure 3:**
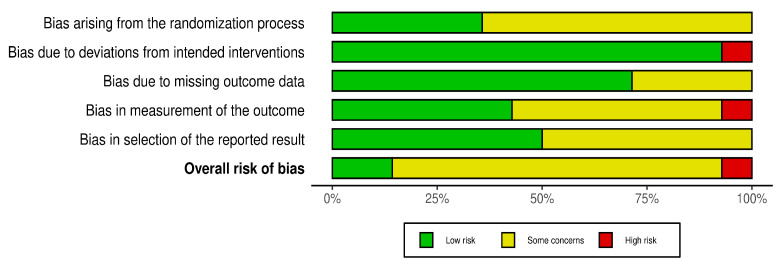
Risk of bias graph.

Only one study was assessed as having a high risk of bias in the domain of deviation from intended intervention (D2) and D4 (outcome measure), indicating isolated but significant problems with adherence to the intervention protocol and potential detection errors. The details of the risk bias are illustrated in Figure 2 and Figure 3.

**Figure 4:**
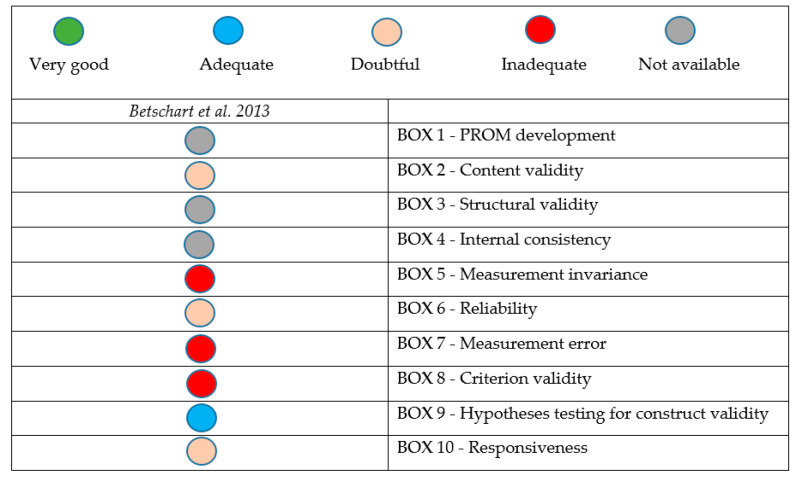
Assessment of study quality and risk of bias based standards for the selection of health measurement instruments (COSMIN) risk of bias tool.

Among the analyzed articles, only one NRCT study was assessed according to COSMIN [26]. The study in question did not receive a “very good” rating in any of the assessed aspects of quality and risk of bias. The highest COSMIN rating concerns hypothesis testing in terms of validity, which was classified as “adequate”. Content validity, reliability, and responsiveness were rated as “doubtful”. However, measurement invariance, measurement error, and criterion validity were indicated as the weakest points and were rated as “inadequate”. The remaining variables were defined as “not available”. Details are presented in Figure 4.

## 4. Discussion

The aim of this systematic review was to analyze training interventions used and their effectiveness in improving pelvic floor muscle function related to urinary incontinence in postmenopausal women. The results of the analyzed studies present a variety of training program proposals, some using additional stimulation. In our review, we qualified for analysis only those studies in which the intervention during the experiment included PFMT, regardless of the proposed form. The vast majority of studies included standard isolated training, while in three studies PFMT was combined with another form of physical activity such as dancing [36], Pilates [37], and Nordic walking [35].

In the scientific literature we can find information on increasing the effectiveness of training by using equipment and applications that enable self-control during exercise [21]. One popular and effective method is biofeedback, thanks to which a woman can monitor the activity of selected muscles in real time [19,20,21,41]. Among the studies qualified for analysis, the use of biofeedback was also observed, after which statistically significant changes in improvement of pelvic floor muscle function were noted. In the study by Pererira et al., vaginal cones were used in one of the groups, which can also be considered a biofeedback mechanism [27,28,42]. Strengthening the pelvic floor muscles using vaginal cones provides sensory-motor biofeedback, which can enhance activation and synchronization of motor units [43]. Another method was used in two of the analyzed studies, in which pelvic floor muscle exercises were used in the form of a virtual video game using a pressure platform [33,39]. The development of technology enables the use of modern equipment and devices for easier acquisition of information about ailments and treatment [44]. In the case of pelvic floor muscle dysfunction, due to limited access to healthcare and the problem of embarrassment for many people, mobile applications are becoming increasingly popular. They enable affordable prevention or therapy of pelvic floor muscle function without side effects and high costs, while motivating the participant of the training program to exercise regularly [45]. In the study conducted by Asklund et al., the mHealth application was used to conduct the training intervention. After three months, significant changes were achieved in reducing urinary incontinence symptoms and improving quality of life [46]. It should be noted that the use of modern self-monitoring equipment may be an appropriate method only for the population that is able to use new technological devices [45]. However, adherence to PFMT may also be limited by physiological barriers such as reduced proprioception, age-related muscle weakening, or a lack of perceived improvement, which often reduces motivation to continue training. These challenges highlight the importance of individualization and appropriate supervision of PFMT interventions [47].

Based on numerous scientific studies, we can find evidence of the positive effect of PFMT on improving pelvic floor muscle function. In our previous studies, we tested the effect of PFMT using biofeedback in the prevention of urinary incontinence symptoms in nulliparous women participating in high-intensity aerobic training [48]. In addition, we conducted a study verifying the effect of a single biofeedback training on improving the technique of pelvic floor muscle exercises in pregnant women [41] and postmenopausal women [20]. We noted positive changes in all studies. In this review, as many as 93% of studies showed statistically significant changes in improving pelvic floor muscle function. Only in one study were no statistically significant changes were observed, but this may be due to too low of a training volume [38]. The researchers decided to perform the exercises on average once every two weeks, which is significantly different from the average of all analyzed studies (3 ± 2 training sessions per week). Currently, it has not been proven which method is the most effective and better than the others, but we know that each form is associated with beneficial changes. Based on the available reports, a combined approach can be proposed, encompassing various forms of intervention individually tailored to the patient [49]. The diversity of PFMT protocols across studies reflects necessary adaptations to specific populations, symptoms, and clinical goals. Factors such as menopausal status, type of pelvic floor dysfunction, and available technology influence the structure and delivery of interventions. This flexibility supports the individualized application of PFMT based on current evidence and therapeutic needs [1,15]. In the absence of a “perfect” solution based on solid evidence, further research is needed to determine the most effective training and the least invasive treatment method [45,49]. However, based on the conducted systematic review, we confirmed that PFMT is an effective method of improving pelvic floor muscle function in postmenopausal women regardless of the specific form of the training intervention. From the perspective of clinicians, including physiotherapists, this means the possibility of flexible selection of the method and form of exercises while maintaining high effectiveness of the therapy. Additionally, the reduction in urinary incontinence symptoms and improvement in quality of life indicate the validity of implementing PFMT as a basic form of conservative treatment in this group of patients. In clinical practice, this may contribute to reducing the need for pharmacotherapy or surgical interventions.

In 14 out of 15 articles analyzed, the prevalence of urinary incontinence was assessed, or one of the questionnaires was used [27,28,29]. Most often, researchers used the 1 h pad test to assess the amount of urine leakage (n = 6) [16,27,28,31,32,36,37] and the International Consultation on Incontinence Questionnaire-Urinary Incontinence Short Form (n = 6) [29,30,36,37,39,40]. Our analyses corresponded with the results of other researchers who obtained similar results [19,50]. From the available questionnaires regarding the assessment of the impact of urinary incontinence on quality of life, the following were also used: Quality of Life [27,28,36]; International Consultation on Incontinence Questionnaire Overactive Bladder [39,40]; Revised Urinary Incontinence Scale [35]; Menopause-Specific Quality of Life Questionnaire [35]; Incontinence Impact Questionnaire Short Form [31]; and UI questionnaire based on a four-point Likert scale [38]. Only one study (7%) did not report any changes in improvement of pelvic floor muscle function. The remaining interventions showed a reduction in the occurrence of urinary incontinence symptoms, and thus an improvement in the quality of life, which is confirmed by the research of Gordon et al. in 2020 [7].

In our analysis, we also paid attention to the detailed description of the training intervention used. A common problem we have identified is the failure to specify all training components necessary for replicating the intervention. Authors often focus on a detailed description of the obtained results, but omit information regarding the description of the frequency, intensity, time, and type of exercise. Inaccurate characterization prevents the implementation of effective training methods by physiotherapists and exercise specialists. In the conducted analysis, a detailed description of all required training components in accordance with ACSM guidelines [22] was recorded in every second publication (53%). We also observed a positive trend that, over the years researchers are increasingly paying attention to ensuring that descriptions are as complete as possible. This may be due to specific standards for describing experimental studies. In a literature review of PFMT in healthy nulliparous women conducted in 2020, full characteristics of the training used were recorded in only 20% of the analyzed studies [51].

### Limitations and Implications

The literature review was conducted using three primary databases: Web of Science, PubMed, and EBSCO (including MEDLINE and SPORTDiscus with Full Text), with no restrictions on publication date. This approach was designed to capture all published studies on applied training interventions aimed at improving pelvic floor muscle function in relation to urinary incontinence among postmenopausal women. Keyword selection was informed by thorough discussions among the coauthors to ensure the inclusion of terminology relevant to the research question. The search strategy combined controlled vocabulary with free-text terms to enhance the breadth and sensitivity of the search. Despite efforts to develop a comprehensive search strategy, it is possible that some relevant studies were missed if the selected keywords did not appear in the titles or abstracts.

Due to language limitations of the research team, only studies in English were included, which could have led to the exclusion of high-quality studies published in other languages, thus introducing a selection bias. Due to the large methodological diversity of the studies qualified for analysis, including the assessment tools used and the different interventions in terms of specific training components, the possibility of conducting a meta-analysis was limited.

The results of this review have several important implications. In terms of practice, they emphasize the need for professionals to tailor interventions based on specific components that demonstrate efficacy, taking into account the characteristics of the target population. There is a need to develop and implement uniform intervention protocols and assessment tools to facilitate effective replication of an effective training program and comparability of results. In addition, more rigorous randomized controlled trials are needed to enable meta-analyses and strengthen the evidence base regarding the effectiveness of training programs in improving pelvic floor muscle function in relation to urinary incontinence in postmenopausal women.

## 5. Conclusions

PFMT is an effective method for improving pelvic floor muscle function in postmenopausal women, regardless of the form of training intervention chosen. Training in a selected form of pelvic floor muscle exercises reduces urinary incontinence symptoms and improves the quality of life of postmenopausal women. In order to enable the implementation of effective interventions, it is necessary to specify in detail all training components that allow for replication of the selected program. In this review, half of the analyzed studies included information on all training components. In addition, patient adherence and proper education regarding the purpose and technique of PFMT are essential to maximize training effectiveness. Incorporating educational strategies into PFMT programs may enhance long-term engagement and treatment effectiveness.

## Figures and Tables

**Table 1:** Characteristics of the analyzed articles.

Author, Year	Characteristics of the Study	Research Tools	Training Effectiveness
Pereira et al. 2012 [28] 2013 [27]	Treatment of stress urinary incontinence, n = 45 G1 (n = 15)—Vaginal cones (age: 66 ± 11; BMI: 27.89 ± 1.93) G2 (n = 15)—PFMT (age: 63 ± 11; BMI: 25.65 ± 2.79) G3 (n = 15)—Control (age: 63 ± 9; BMI: 26.04 ± 1.84)	(a) 1 h pad test (b) perineometer (c) questionnaires: QoL KHQ	Statistically significant improvement in both intervention groups (G1 and G2) in both decreased urine leakage and increase in PFM pressure. Both groups noticed an improvement in the quality of life.
Betschart et al. 2013 [38]	Treatment of urinary incontinence, n = 120 G1 (n = 41)—Premenopausal women (PFMT) (age: 41 ± 4; BMI: 27.0 ± 6.8) G2 (n = 79)—Postmenopausal women (PFMT) (age: 74 ± 6; BMI: 27.4 ± 5.6)	UI questionnaire based on a four-point Likert scale	There was a noticeable improvement in both groups, and no differences were observed between the groups. Changes were not statistically significant.
Alves et al. 2015 [29]	Treatment of urinary incontinence, n = 42 G1 (n = 18)—Treatment (PFMT) (age: 66 ± 9; BMI: 29.43 ± 3.91) G2 (n = 12)—Control (age: 66 ± 9; BMI: 31.52 ± 5.71)	(a) digital palpation; (b) sEMG assessment; questionnaires: ICIQ-UI SF	(a–c) Statistically significant improvement in G1
Botelho et al. 2015a [40]	Improving the function of the PFM, (PFMT) n = 82 G1 (n = 11)—Nulliparous women (age: 25 ± 4) G2 (n = 13)—Primiparous pregnant (age: 23 ± 6) G3 (n = 20)—Primiparous postpartum (age: 23 ± 6) G4 (n = 38)—Postmenopausal women (age: 65 ± 8)	(a) digital palpation; (b) sEMG or vaginal dynamometry; (c) questionnaires: ICIQ-UI SF; ICIQ-OAB	Statistically significant improvement in all intervention groups in both decreased urine leakage and increase in PFM pressure. All groups noticed an improvement in their quality of life.
Botelho et al. 2015b [39]	Improving the function of the PFM, n = 46 (video game on a pressure base platform) G1 (n = 19)—Nulliparous women without urinary incontinence (age: 25 ± 4) G2 (n = 27)—Postmenopausal women with urinary incontinence (age: 62 ± 8)	(a) G1: digital palpation; sEMG G2: vaginal dynamometry and digital palpation; (b) questionnaires: ICIQ-UI SF; ICIQ-OAB	In G1, a significant increase in strength was noted on palpation, which was not confirmed by sEMG. In G2, there were no changes in digital palpation, but there was improvement in dynamometer assessment and questionnaires.
Castellani et al. 2015 [31]	Treatment of urinary incontinence, n = 69 G1 (n = 35)—PFMT + ES + BF (age: 55 ± 5; BMI: 25.2 ± 2.1) G2 (n = 34)—PFMT + ES + BF + 1 mg intravaginal estriol ovule once daily for 4 weeks and then two ovules once weekly for 20 weeks (age: 55 ± 6; BMI: 24.7 ± 2.2)	(a) UDS/VLPP 1 h pad test (b) questionnaires: IIQ-7	(a) Symptoms scores and incontinence status were statistically significant better in G2; (b) Statistically significant improvement in both groups.
Tosun et al. 2015 [32]	Treatment of urinary incontinence, n = 103 G1 (n = 41)—Late reproductive age/early transitional (age: 46 ± 6; BMI: 30.23 ± 6.64) G2 (n = 32)—Final menstrual bleeding 2–5 years ago (age: 52 ± 5; BMI: 30.55 ± 5.5) G3 (n = 30)—Final menstrual bleeding more than 5 years ago (age: 67 ± 7; BMI: 27.06 ± 5.0)	1 h pad test; PERFECT scheme; Brink scale; perineometry; ultrasound	Statistically significant increase in PERFECT scheme, Brink scale, perineometry, and ultrasound values among the three groups.
Martinho et al. 2016 [33]	Improving the function of the PFM, n = 47 G1 (n = 27)—Abdominopelvic training by virtual reality (age: 62 ± 9; BMI: 28.1 ± 3.9) G2 (n = 20)—PFMT using by gym ball (age: 62 ± 9; BMI: 28 ± 3.7)	digital palpation; vaginal dynamometry	Only the endurance parameter showed a significant difference between groups, given that the G1 had a significant improvement after training, while the G2 had a significant decrease in the same parameter.
Bertotto et al. 2017 [34]	Treatment of urinary incontinence, n = 45 G1 (n = 15)—PFMT (age: 59 ± 5; BMI: 27.7 ± 3.6) G2 (n = 16)—PFMT + biofeedback (age: 58 ± 7; BMI: 27.5 ± 2.6) G3 (n = 14)—Control group (age: 57 ± 5; BMI: 26.8 ± 3.6)	digital palpation; sEMG + biofeedback; questionnaire: QoL	Significant improvement in both intervention groups. The duration of endurance contraction and MVC showed significantly greater improvement in G2.
Antônio et al. 2018 [30]	Improving the function of the PFM, n = 99 G1 * (n = 51)—PFMT (age: 53 ± 4; BMI: 28.5 ± 5.4) G2 * (n = 48)—Control group (age: 53 ± 4; BMI: 28.3 ± 4.8) * both subgroups included women using hormone therapy	(a) manometry (b) questionnaire: ICIQ-UI SF	(a,b) Statistically significant improvement in subgroups not using hormone therapy.
Elbandrawy et al. 2022 [35]	Effect of aerobic walking exercise on stress urinary incontinence, n = 30 G1 (n = 15)—PFMT (age: 56 ± 3; BMI: 26.9 ± 1.8) G2 (n = 15)—PFMT in addition to aerobic walking exercise (age: 53 ± 4; BMI: 28.5 ± 5.4)	(a) sEMG (b) questionnaire: RUIS	(a,b) Statistically significant improvement in G2.
Kolodynska et al. 2022 [16]	Treatment of stress urinary incontinence, n = 60 (age: 57 ± 6; BMI: 27.07 ± 4.69): G1 (n = 20)—Sonofeedback G2 (n = 20)—ES +BF G3 (n = 20)—Control	(a) Gaudenz questionnaire (b) modified 1 h pad test	(a + b) Statistically significant improvement in G2. During laughter and coughing, there was a significant reduction in urine leakage in G1.
Tang et al. 2023 [36]	Treatment of stress urinary incontinence, n = 24 G1 (n = 13)—Rumba dance combined with breathing training (age: 53 ± 6; BMI: 23.8 ± 2.9) G2 (n = 11)—Control group (age: 57 ± 5; BMI: 23.3 ± 2.9)	(a) 1 h pad test (b) VRP (c) questionnaire: ICIQ-UI SF; QoL	(a–c) Statistically significant improvement in G1.
Gonzaga et al. 2024 [37]	Treatment of stress urinary incontinence, n = 40 G1 (n = 20)—PFMT G2 (n = 20)—Pilates	(a) 1 h pad test (b) manometry (c) questionnaire: ICIQ-UI SF	(a,c) Statistically significant improvement in both groups. (b) Peak strength manometry was significantly improved only in the Pilates group, and the mean strength manometry in both groups.
Elgayar, 2024 [17]	Improving the function of the PFM, n = 43 G1 (n = 22)—HIFEMT + PFMT (age: 50 ± 5; BMI: 26.3 ± 2.9) G2 (n = 21)—Control (Only PFMT) (age: 51 ± 4; BMI: 25.3 ± 2.8)	(a) perineometer (b) questionnaire: MENQOL	(a,b) Statistically significant improvement in both groups. Significantly greater changes were noted in G1.

BF—biofeedback; BMI—body mass index; ES—electrical stimulation; G—group; HIFEMT—high-intensity focused electromagnetic therapy; ICIQ-OAB—International Consultation on Incontinence Questionnaire Overactive Bladder; ICIQ-UI SF—International Consultation on Incontinence Questionnaire-Urinary Incontinence Short Form; IIQ-7—Incontinence Impact Questionnaire Short Form; KHQ—King’s Health Questionnaire; MENQOL—menopause-specific quality of life questionnaire; MVC—maximal voluntary contraction; PFM—pelvic floor muscle; PFMT—pelvic floor muscle training; RUIS—Revised Urinary Incontinence Scale; QoL—quality of life; sEMG—surface electromyography; UI—urinary incontinence; UDS—urodynamics; VLPP—Valsalva leak point pressure; VRP—vaginal resting pressure; * use of hormone therapy.

**Table 2:** Characteristics of the PFMT components used in the analyzed studies.

Author, Year	Type (Description of Exercise) Contraction/Rest (s.)	Intensity of PFM Contraction	Frequency (n Times per Week)	Duration/Time of a Single Session (Minutes/Repetitions)	Duration of Entire Program (Number of Weeks/Sessions)
Pereira et al. 2012 [28] 2013 [27]	3 s./6 s. 5-10 s./10–20 s.	maximum contraction	2	40′ 100 repetitions	6 weeks (12 sessions)
Betschart et al. 2013 [38]	-	-	on average once every two weeks	30–45′	3 months (5–9 sessions)
Alves et al. 2015 [29]	8 s./16 s.	maximum contraction	2	30′ four sets of 10 repetitions of each exercise	6 weeks (12 sessions)
Botelho et al. 2015a [40]	Quick flicks; 8 s./16 s.	maximum contraction	3	60′ four sets of 10 repetitions of each exercise	10 sessions
Botelho et al. 2015b [39]	Video game: pelvic anteversion, retroversion, lateral tilting, and circumduction movements.	-	2	30′	5 weeks (10 sessions)
Castellani et al. 2015 [31]	5 s./5 s. 2 s./2 s. 1 s./1 s. 10 s./10 s.	-	2	30′ 10 repetitions 20 repetitions 20 repetitions 5 repetitions	24 weeks (48 sessions)
Tosun et al. 2015 [32]	-	-	3	30′	12 weeks (36 sessions)
Martinho et al. 2016 [33]	G1—Video game: 300 s./90 s. pelvic anteversion, retroversion, lateral tilting, and circumduction movements.	-	2	30′ four sets	5 weeks (10 sessions)
	G2: quick flicks 8 s./16 s.			four sets four sets	
Bertotto et al. 2017 [34]	6-10 s./6–10 s. 2 s./4 s. 3-5 s./6–10 s.	maximum contraction	2	20′ 10 repetitions; two sets	4 weeks (8 sessions)
Antônio et al. 2018 [30]	6 s./6 s. Quick flicks/6 s.	maximum contraction	2—therapy 5—home	10 repetitions	12 weeks (84 sessions)
Elbandrawy et al. 2022 [35]	6-8 s./6 s. 3-4 quick flicks/6 s.	maximum contraction	2—therapy	8–12 repetitions	12 weeks (84 sessions)
	3–10 s.		5—home	five sets of 10 repetitions	
Kolodynska et al. 2022 [16]	5 s./10 s.	maximum contraction G2—max. Current up to 100 mA (1 ms)	5	30′ 10 sets of 10 repetitions	2 weeks (10 sessions)
Tang et al. 2023 [36]	Rumba dance combined with breathing training	-	3	90′: 5′ warm-up 20′ abdominal breathing exercise 60′ Rumba dance 5′ relaxation	16 weeks (48 sessions)
Gonzaga et al. 2024 [37]	PFMT/Pilates	maximum contraction	3	-	12 weeks (36 sessions)
Elgayar, 2024 [17]	Quick flicks/6 s.	maximum contraction	2	Three sets of 8–12 repetitions	12 weeks (24 sessions)

## Data Availability

The raw data supporting the conclusions of this article will be made available by the authors on request.

## Abbreviations

The following abbreviations are used in this manuscript:

BF	Biofeedback
BMI	Body mass index
ES	Electrical stimulation
G	Group
HIFEMT	High-intensity focused electromagnetic therapy
ICIQ-OAB	International Consultation on Incontinence Questionnaire Overactive Bladder
ICIQ-UI-SF	International Consultation on Incontinence Questionnaire-Urinary Incontinence Short Form
IIQ-7	Incontinence Impact Questionnaire Short Form
KHQ	King’s Health Questionnaire
MENQOL	Menopause-specific quality of life questionnaire
MVC	Maximal voluntary contraction
NRCT	Non-randomized controlled trial
PFM	Pelvic floor muscle
PFMT	Pelvic floor muscle training
RCT	Randomized controlled trial
RoB	Risk of bias
RUIS	Revised Urinary Incontinence Scale
QoL	Quality of life
sEMG	Surface electromyography
UI	Urinary incontinence
UDS	Urodynamics
VLPP	Valsalva leak point pressure
VRP	Vaginal resting pressure

## References

[1] Bo K., Frawley H.C., Haylen B.T., Abramov Y., Almeida F.G., Berghmans B., Bortolini M., Dumoulin C., Gomes M., McClurg D. (2017). An International Urogynecological Association (IUGA)/International Continence Society (ICS) joint report on the terminology for the conservative and nonpharmacological management of female pelvic floor dysfunction. Int. Urogynecol. J..

[2] Sam P., LaGrange C. Anatomy, Abdomen and Pelvis, Penis. https://www.ncbi.nlm.nih.gov/books/NBK482236/.

[3] Omodei M.S., Marques Gomes Delmanto L.R., Carvalho-Pessoa E., Schmitt E.B., Nahas G.P., Petri Nahas E.A. (2019). Association Between Pelvic Floor Muscle Strength and Sexual Function in Postmenopausal Women. J. Sex. Med..

[4] Zhuo Z.H., Wang C.H., Yu H.M., Li J. (2021). The Relationship Between Pelvic Floor Function and Sexual Function in Perimenopausal Women. Sex. Med..

[5] Okeahialam N.A., Dworzynski K., Jacklin P., McClurg D. (2022). Prevention and non-surgical management of pelvic floor dysfunction: Summary of NICE guidance. BMJ.

[6] Mottola M.F., Davenport M.H., Ruchat S.M., Davies G.A., Poitras V.J., Gray C.E., Jaramillo Garcia A., Barrowman N., Adamo K.B., Duggan M. (2018). 2019 Canadian guideline for physical activity throughout pregnancy. Br. J. Sports Med..

[7] Gordon S., Ruivo D.B., Viscardi L.G.A., Oliveira A.S.d. (2020). Effects of the Pilates method isolated and associated with manual therapy in women with urinary incontinence. Man. Ther. Posturology Rehabil. J..

[8] Abufaraj M., Xu T., Cao C., Siyam A., Isleem U., Massad A., Soria F., Shariat S.F., Sutcliffe S., Yang L. (2021). Prevalence and trends in urinary incontinence among women in the United States, 2005–2018. Am. J. Obs. Gynecol..

[9] Milsom I., Gyhagen M. (2019). The prevalence of urinary incontinence. Climacteric.

[10] El Khoudary S.R., Greendale G., Crawford S.L., Avis N.E., Brooks M.M., Thurston R.C., Karvonen-Gutierrez C., Waetjen L.E., Matthews K. (2019). The menopause transition and women’s health at midlife: A progress report from the Study of Women’s Health Across the Nation (SWAN). Menopause-J. N. Am. Menopause Soc..

[11] Prajapati M.M. (2020). Awareness regarding menopausal symptoms and effect on daily life among postmenopausal women. J. Patan Acad. Health Sci..

[12] Seyyedi F., Rafiean-Kopaei M., Miraj S. (2016). Comparison of the Effects of Vaginal Royal Jelly and Vaginal Estrogen on Quality of Life, Sexual and Urinary Function in Postmenopausal Women. J. Clin. Diagn. Res..

[13] Oplawski M., Smoczynska M., Grabarek B.O., Boron D. (2021). Assessment of Dysfunction in the Urinary System as Well as Comfort in the Life of Women during and after Combination Therapy Due to Ovarian and Endometrial Cancer Based on the SWL, II-Q7 and UDI-6 Scales. J. Clin. Med..

[14] KEGEL A. (1948). Progressive resistance exercise in the functional restoration of the perineal muscles. Am. J. Obstet. Gynecol..

[15] Hay-Smith E.J., LC B.B., Hendriks H.J., de Bie R.A., van Waalwijk van Doorn E.S. (2008). Pelvic floor muscle training for urinary incontinence in women. Cochrane Database Syst. Rev..

[16] Kolodynska G., Zalewski M., Mucha A., Andrzejewski W. (2022). Assessment of the Effectiveness of the Sonofeedback Method in the Treatment of Stress Urinary Incontinence in Women-Preliminary Report. J. Clin. Med..

[17] Elgayar S. (2024). Combined effects of high-intensity focused electromagnetic therapy and pelvic floor exercises on pelvic floor muscles and sexual function in postmenopausal women. Obstet. Gynecol. Sci..

[18] Campanini I., Disselhorst-Klug C., Rymer W.Z., Merletti R. (2020). Surface EMG in Clinical Assessment and Neurorehabilitation: Barriers Limiting Its Use. Front. Neurol..

[19] Hagen S., Elders A., Stratton S., Sergenson N., Bugge C., Dean S., Hay-Smith J., Kilonzo M., Dimitrova M., Abdel-Fattah M. (2020). Effectiveness of pelvic floor muscle training with and without electromyographic biofeedback for urinary incontinence in women: Multicentre randomised controlled trial. BMJ-Br. Med. J..

[20] Piernicka M., Ossowski Z., Kortas J., Bojar D., Labun J., Szumilewicz A. (2024). Can We Improve the Technique of Pelvic Floor Muscle Exercises in Postmenopausal Women Using a Single Electromyography Biofeedback Session? An Experimental Study. J. Clin. Med..

[21] Romeikienė K.E., Bartkevičienė D. (2021). Pelvic-Floor Dysfunction Prevention in Prepartum and Postpartum Periods. Medicina.

[22] Medicine A.C.o.S. (2017). ACS’M Resources for the Personal Trainer.

[23] Page M., McKenzie J., Bossuyt P., Boutron I., Hoffmann T., Mulrow C., Shamseer L., Tetzlaff J., Akl E., Brennan S. (2021). The PRISMA 2020 statement: An updated guideline for reporting systematic reviews. Syst. Rev..

[24] Sterne J., Savovic J., Page M., Elbers R., Blencowe N., Boutron I., Cates C., Cheng H., Corbett M., Eldridge S. (2019). RoB 2: A revised tool for assessing risk of bias in randomised trials. BMJ-Br. Med. J..

[25] McGuinness L., Higgins J. (2021). Risk-of-bias VISualization (robvis): An R package and Shiny web app for visualizing risk-of-bias assessments. Res. Synth. Methods.

[26] Mokkink L., Boers M., van der Vleuten C., Bouter L., Alonso J., Patrick D., de Vet H., Terwee C. (2020). COSMIN Risk of Bias tool to assess the quality of studies on reliability or measurement error of outcome measurement instruments: A Delphi study. BMC Med. Res. Methodol..

[27] Pereira V.S., de Melo M.V., Correia G.N., Driusso P. (2013). Long-term effects of pelvic floor muscle training with vaginal cone in post-menopausal women with urinary incontinence: A randomized controlled trial. Neurourol. Urodyn..

[28] Pereira V., de Melo M., Correia G., Driusso P. (2012). Vaginal cone for postmenopausal women with stress urinary incontinence: Randomized, controlled trial. Climacteric.

[29] Alves F., Riccetto C., Adami D., Marques J., Pereira L., Palma P., Botelho S. (2015). A pelvic floor muscle training program in postmenopausal women: A randomized controlled trial. Maturitas.

[30] Antônio F., Herbert R., Bo K., Rosa-e-Silva A., Lara L., Franco M., Ferreira C. (2018). Pelvic floor muscle training increases pelvic floor muscle strength more in post-menopausal women who are not using hormone therapy than in women who are using hormone therapy: A randomised trial. J. Physiother..

[31] Castellani D., Saldutto P., Galica V., Pace G., Biferi D., Galatioto G., Vicentini C. (2015). Low-Dose Intravaginal Estriol and Pelvic Floor Rehabilitation in Post-Menopausal Stress Urinary Incontinence. Urol. Int..

[32] Tosun Ö., Mutlu E., Tosun G., Ergenoglu A., Yeniel A., Malkoç M., Askar N., Itil I. (2015). Do stages of menopause affect the outcomes of pelvic floor muscle training?. Menopause-J. N. Am. Menopause Soc..

[33] Martinho N., Silva V., Marques J., Carvalho L., Iunes D., Botelho S. (2016). The effects of training by virtual reality or gym ball on pelvic floor muscle strength in postmenopausal women: A randomized controlled trial. Braz. J. Phys. Ther..

[34] Bertotto A., Schvartzman R., Uchôa S., Wender M. (2017). Effect of electromyographic biofeedback as an add-on to pelvic floor muscle exercises on neuromuscular outcomes and quality of life in postmenopausal women with stress urinary incontinence: A randomized controlled trial. Neurourol. Urodyn..

[35] Elbandrawy A.M., Mahmoud S.G., AboElinin M.F., Yousef A.M. (2022). Effect of Aerobic Walking Exercise on Stress Urinary Incontinence in Postmenopausal Women. Women Sport. Phys. Act. J..

[36] Tang Y., Guo X., Wang Y., Liu Z., Cao G., Zhou Y., Chen M., Liu J., Mu J., Yuan M. (2022). Rumba Dance Combined with Breathing Training as an Exercise Intervention in the Management of Stress Urinary Incontinence in Postmenopausal Women: A Randomized Controlled Trial. Int. J. Environ. Res. Public Health.

[37] Gonzaga S., de Oliveira R., Dutra L., Oliveira L., de Oliveira L. (2024). Comparative analysis of pelvic floor muscle training and Pilates in managing urinary incontinence among postmenopausal women: A randomized controlled trial. Int. Urogynecol. J..

[38] Betschart C., Mol S., Lütolf-Keller B., Fink D., Perucchini D., Scheiner D. (2013). Pelvic Floor Muscle Training for Urinary Incontinence: A Comparison of Outcomes in Premenopausal Versus Postmenopausal Women. Female Pelvic Med. Reconstr. Surg..

[39] Botelho S., Martinho N., Silva V., Marques J., Carvalho L., Riccetto C. (2015). Virtual reality: A proposal for pelvic floor muscle training. Int. Urogynecol. J..

[40] Botelho S., Martinho N., Silva V., Marques J., Alves F., Riccetto C. (2015). Abdominopelvic kinesiotherapy for pelvic floor muscle training: A tested proposal in different groups. Int. Urogynecol. J..

[41] Bludnicka M., Piernicka M., Kortas J., Biernacka B.D., Szumilewicz A. (2020). Effects of a One-Time Biofeedback EMG Session on Neuromuscular Activity of the Pelvic Floor Muscles in Pregnant Women. Neurophysiology.

[42] Villani F., Petre I., Buleu F., Iurciuc S., Marc L., Apostol A., Valentini C., Donati E., Simoncini T., Furau C. (2024). Pelvic Floor Muscle Training vs. Vaginal Vibration Cone Therapy for Postpartum Dyspareunia and Vaginal Laxity. Medicina.

[43] Gameiro M.O., Moreira E.H., Gameiro F.O., Moreno J.C., Padovani C.R., Amaro J.L. (2010). Vaginal weight cone versus assisted pelvic floor muscle training in the treatment of female urinary incontinence. A prospective, single-blind, randomized trial. Int. Urogynecol. J..

[44] Hamine S., Gerth-Guyette E., Faulx D., Green B.B., Ginsburg A.S. (2015). Impact of mHealth chronic disease management on treatment adherence and patient outcomes: A systematic review. J. Med. Internet Res..

[45] -Ahmed R., Taithongchai A., da Silva A., Robinson D., Cardozo L. (2023). Treating and Managing Urinary Incontinence: Evolving and Potential Multicomponent Medical and Lifestyle Interventions. Res. Rep. Urol..

[46] Asklund I., Nyström E., Sjöström M., Umefjord G., Stenlund H., Samuelsson E. (2017). Mobile app for treatment of stress urinary incontinence: A randomized controlled trial. Neurourol. Urodyn..

[47] Harper R., Sheppard S., Stewart C., Clark C. (2023). Exploring Adherence to Pelvic Floor Muscle Training in Women Using Mobile Apps: Scoping Review. JMIR Mhealth Uhealth.

[48] Piernicka M., Bludnicka M., Kortas J., Duda-Biernacka B., Szumilewicz A. (2021). High-impact aerobics programme supplemented by pelvic floor muscle training does not impair the function of pelvic floor muscles in active nulliparous women A randomized control trial. Medicine.

[49] Bapir R., Bhatti K.H., Eliwa A., García-Perdomo H.A., Gherabi N., Hennessey D., Magri V., Mourmouris P., Ouattara A., Perletti G. (2023). Treatment of urge incontinence in postmenopausal women: A systematic review. Arch. Ital. di Urol. e Androl..

[50] Woodley S.J., Boyle R., Cody J.D., Morkved S., Hay-Smith E.J.C. (2017). Pelvic floormuscle training for prevention and treatment of urinary and faecal incontinence in antenatal and postnatal women. Cochrane Database Syst. Rev..

[51] Piernicka M., Duda-Biernacka B., Bludnicka M., Szumilewicz A. (2020). The Characteristics of the Pelvic Floor Muscle Training Programs Used in Experimental Studies with Surface Electromyography in Non-Pregnant Women: A Systematic Review. Iran. J. Public Health.

